# Relationship Quality With Adult Children Among Grandparent Caregivers

**DOI:** 10.1093/geroni/igaa057.245

**Published:** 2020-12-16

**Authors:** Heejung Jang

**Affiliations:** University of Michigan, Ann Arbor, Michigan, United States

## Abstract

For the increasing number of grandparent caregivers, relationship quality with adult children has important implications for the well-being of grandparents. Based on solidarity, conflict, and ambivalence, the present study examines how parent-adult children's relationships differ by grandparent caregiving status on depressive symptoms and psychological well-being. This study uses The 2014 Health and Retirement Study from a sample of 1,197 grandparent caregivers age 51 and older. Latent class analysis is applied to measure affection and conflict in older grandparents-adult children relationships. Results from the latent class analysis identified four clusters: amicable, ambivalent, detached, and disharmonious. OLS regression models are estimated the association between relationship types and depressive symptoms and psychological well-being by grandparent caregiving. For the depressive symptoms, disharmonious relationships with adult children increase depressive symptoms among co-parenting and custodial grandparents. Also, ambivalent and disharmonious relationships with adult children reduce the psychological well-being of older grandparents. The study discusses the variances of the relationships with adult children and their effects on grandparents’ well-being. The results will shed light on the importance of familial relationships and will be beneficial for the development and maintenance of policies and practices that support the families of grandparent caregivers.

